# Phase I/II trial of capecitabine, oxaliplatin, and irinotecan in combination with bevacizumab in first line treatment of metastatic colorectal cancer

**DOI:** 10.1002/cam4.497

**Published:** 2015-07-24

**Authors:** Shouki Bazarbashi, Ali Aljubran, Ahmad Alzahrani, Ahmed Mohieldin, Hussein Soudy, Mohammed Shoukri

**Affiliations:** 1 Oncology Center, King Faisal Specialist Hospital and Research CenterPO Box 3354, Riyadh, 11211, Saudi Arabia; 2 Medical Oncology Department, Zagazig UniversityAl-Gamaá Road, Zagazig, Sharkia Governorate, 44519, Egypt; 3 Faculty of medicine, Cairo UniversityKasr Al-Ainy Street, Cairo, 11562, Egypt; 4 Department of Biostatistics, Epidemiology and Scientific Computing Research Center, King Faisal Specialist Hospital and Research CenterPO Box 3354, Riyadh, 11211, Saudi Arabia

**Keywords:** Bevacizumab, capecitabine, irinotecan, metastatic colorectal cancer, oxaliplatin

## Abstract

Phase III studies have demonstrated the efficacy of FOLFOXIRI regimens (5-fluorouracil/leucovorin, oxaliplatin, irinotecan) with/without bevacizumab in metastatic colorectal cancer (mCRC). Capecitabine is an orally administered fluoropyrimidine that may be used instead of 5-fluorouracil/leucovorin. We evaluated a triple-chemotherapy regimen of capecitabine, oxaliplatin, and irinotecan, plus bevacizumab in 53 patients with mCRC. A Phase I study identified the maximum tolerated dose of irinotecan as 150 mg/m^2^. Median follow-up in a subsequent Phase II study using this dose was 28 months (74% progressed). For all patients, a complete response was achieved in 4% and a partial response in 60%; median progression-free survival (PFS) was 16 months and median overall survival (OS) was 28 months. Median PFS was longer for patients with an early treatment response (28 vs. 9 months for others; *P* = 0.024), or early tumor shrinkage (25 vs. 9 months for others; *P* = 0.006), or for patients suitable for surgical removal of metastases with curative intent (median not reached vs. 9 months for others; *P* = 0.001). Median OS was longer for patients with early tumor shrinkage (median not reached vs. 22 months for others; *P* = 0.006) or surgery (median not reached vs. 22 months for others, *P* = 0.002). K-ras mutations status did not influence PFS (*P* = 0.88) or OS (*P* = 0.82). Considerable Grade 3/4 toxicity was encountered (36% for diarrhea, 21% for vomiting and 17% for fatigue). In conclusion, the 3-weekly triple-chemotherapy regimen of capecitabine, oxaliplatin, and irinotecan, plus bevacizumab, was active in the first-line treatment of mCRC, although at the expense of a high level of toxicity.

## Introduction

Colorectal cancer (CRC) is the third most commonly diagnosed cancer in males and the second most common in females; more than 1.2 million new cases and 608,700 deaths have occurred worldwide in 2008. 1 Chemotherapy remains the primary therapeutic option for patients with metastatic CRC (mCRC). Combinations of fluoropyrimidines, irinotecan, and oxaliplatin have been shown to be effective in this setting, along with the more recent introduction of targeted chemotherapy with monoclonal antibodies against the vascular endothelial growth factor (bevacizumab) or the epidermal growth factor receptor (cetuximab and panitumumab). The addition of a targeted agent to first line chemotherapy has improved progression free survival (PFS) and overall survival (OS) in randomized trials 2–6 and is now considered to represent the standard of care for mCRC.

Phase III trials have shown the FOLFOXIRI (5-flourouracil [5-FU]/leucovorin, oxaliplatin, irinotecan) to be superior to the FOLFIRI (5-FU/leucovorin, irinotecan) regimen, in terms of improvements in response rate, PFS, and OS, with or without addition of bevacizumab 7,8. This improved efficacy was at the expense of increased toxicity which was manageable, as the incidence of grade 3 or 4 toxicity did not exceed 20% 7–9. Nevertheless, infusional chemotherapy requires a central venous catheter with its associated complications, frequent hospital visits (every 2 weeks) and the need to carry a pump. Capecitabine, an oral fluoropyrimidine, was at least as active and effective as 5-FU in the first-line treatment of mCRC in Phase III trials, with a superior safety profile 10–12. Similarly, a combination of capecitabine and oxaliplatin (Xelox or Capox) but not irinotecan (Xeliri/Capiri) was at least as effective as its infusional counterpart, FOLFOX 13–19.

It is therefore of interest to study the therapeutic profile of capecitabine within a triple-therapy regimen, with the addition of targeted chemotherapy in mCRC. Accordingly, we have evaluated the combination of capecitabine, oxaliplatin, and irinotecan with bevacizumab in the first-line management of mCRC in a Phase I/II trial.

## Materials and Methods

### Patients

The study was conducted at a single institution. Patients eligible for inclusion in the study were men or women aged ≥18 years with histologically confirmed colorectal adenocarcinoma presenting as unresectable metastatic or locally advanced disease; Eastern Cooperative Oncology Group performance status (ECOG PS) of 0–2; measurable disease as defined by Response Evaluation Criteria in Solid Tumors (RECIST); no previous chemotherapy or bevacizumab for metastatic disease; and adequate hematological, renal, and hepatic function (absolute neutrophil count ≥1.5 × 10^9^/L, platelet count ≥100 × 10^9^/L, normal serum creatinine, normal serum bilirubin, serum transaminases ≤2.5 times the upper limit of normal [UNL; ≤5.0 times UNL if elevated secondary to liver metastases]); and urine dipstick for protinuria <2+. Patients who had received prior adjuvant 5-FU or oxaliplatin chemotherapy were eligible if they had remained free of disease for at least 12 months after the completion of adjuvant therapy.

Exclusion criteria included patients with known or suspected dihydropyrimidine deficiency; the presence of central nervous system metastasis; previous malignancy within the last 5 years (except adequately treated nonmelanomatous skin cancer or in situ cervical cancer); severe cardiovascular disease; major bleeding disorder; significant traumatic injury or major surgery within 28 days of starting therapy; minor surgery within 7 days of starting therapy; recent significant hemoptysis; active uncontrolled infection; uncontrolled hypertension; pregnancy or breastfeeding; any other serious medical condition (in the judgment of the investigator); treatment with other experimental drugs within 30 days of entry into the trial; treatment with other anticancer therapy; known hypersensitivity to any of the study drugs; any psychological, familial, geographic, or social circumstances which could impair the patient's ability to participate in the trial and comply with follow-up, including legal incapacity.

### Treatment

Pretreatment baseline evaluation included a complete medical history and physical examination, full blood count and chemistry profile including carcinoembryonic antigen, and a CT scan of the chest, abdomen, and pelvis.

The Phase I trial was designed to find the maximum tolerated dose of irinotecan, with a design based on the standard 3-week Xelox/Capox regimen, in order to minimize the requirements for hospital attendance while maintaining efficacy. All patients received oral capecitabine 1000 mg/m^2^ twice-daily on days 1–14, with intravenous oxaliplatin 130 mg/m^2^, and bevacizumab 7.5 mg/kg body weight on day 1.

The prespecified dose levels for irinotecan were 150, 200, and 250 mg/m^2^, given intravenously on day one of each cycle. The starting dose was based on previous clinical experience with the use of irinotecan within three-drug combinations, which involved administration of this agent at starting doses of 150–180 mg/m^2^ given every 2 weeks 20,21. Accordingly, we adopted the dose of 150 mg/m^2^ as a starting dose to explore the maximum tolerated dose within the more standard three-weekly administration regimen employed here.

At least three patients were included sequentially in each dose level, and no intrapatient dose-escalation was allowed. Dose escalation was permitted if no dose limiting toxicity (any grade 4 hematological toxicity and/or grade 3 or 4 nonhematological toxicity) was encountered by the end of the first cycle. If one of three patients experienced dose-limiting toxicity, three additional patients were enrolled at the same dose level. The maximum tolerated dose was defined according to the occurrence of dose-limiting toxicity in least 2/3 or at least 4/6 patients.

The recommended dose for the Phase II study was the dose level immediately below the maximum tolerated dose. Additional patients were then enrolled to confirm the safety profile of the combination 22. Treatment was administered every 21 days. A total of 5–8 cycles of the four drug combination was planned, followed by maintenance capecitabine and bevacizumab at the same dose level until disease progression.

During either phase, the feasibility of surgical resection of metastatic sites was assessed every 2 months and strongly recommended when feasible. Treatment was withdrawn in the event of disease progression, unacceptable toxicity, or withdrawal of patient consent.

### Dose modification

Dose modifications were made according to the most serious toxicity observed during the previous cycle, graded according to the National Cancer Institute Common Terminology Criteria (version 3) 23. Only the capecitabine dose was modified for hand-foot syndrome and mucositis, the capecitabine, and irinotecan doses could be modified for diarrhea, and the oxaliplatin was modified for neuropathy. Bevacizumab doses were not modified. Chemotherapy treatment was delayed until neutrophil count was ≥1.0 × 10^9^/L and platelet count was ≥100 × 10^9^/L prior to start of the next cycle. Patients were withdrawn from the trial if toxicity required treatment to be delayed by more than 2 weeks.

### Study endpoints

The primary endpoint for the Phase I study was to identify the maximum tolerated dose of irinotecan. Primary outcomes for the Phase II study were the response rate and toxicity profile in patients with mCRC. Secondary endpoints were PFS and OS. PFS was calculated from the day of treatment start to the first observation of disease progression or death from any cause. OS was calculated from the day of treatment start until death from any cause, censoring patients where necessary at the last date known to be alive.

### Evaluation of response

Assessment of response was done according to RECIST criteria, version 1.1 24 A CT scan or MRI scan of the chest, abdomen, and pelvis were done after the second, fifth and eighth cycle of chemotherapy and then every 2 months. This schedule facilitated detection of early tumor shrinkage after the first 6 weeks, followed by subsequent regular, two-monthly evaluation. Deepness of response (DpR) was defined as the percentage of tumor shrinkage observed (if shrinkage occurred) at the nadir (best response) using the longest diameter based on RECIST criteria 25. Early tumor shrinkage was defined as ≥20% decrease in the maximum tumor dimension by RECIST criteria at the time of first evaluation of response 26.

### Follow-up and end of study visits

All patients underwent measurement of complete blood count with differential, renal, and hepatic profile, carcino-emberyonic antigen (CEA), and urine for proteinuria on day one of each cycle. Blood count was also done on the day 10–14 for the first two cycles. Toxicity evaluation was recorded prior to starting treatment and on day 1 of each cycle of chemotherapy. Patients were followed up until the study was closed upon reaching the planned number of events.

### Statistics

The number of patients to be recruited in the Phase I trial was depended on the maximum tolerated dose of irinotecan. The number of patients for the Phase II trial followed a two-stage Simon optima design to include the patient recruited in Phase I. For a lower activity level of 40% (*P*_0_=0.40, percentage of patients free of progression at 10 months in the null hypothesis) and higher activity level of 60% (*P*_1_=0.60, percentage of patients free of progression at 10 months in the alternative hypothesis), and with *α* and *β* error of 0.05 and 0.20, the Phase I trial was planned to recruit 16 patients. If fewer than seven patients achieved an objective response, the trial would close as the study treatment was not more effective than standard chemotherapy. If more than seven patients in Phase I achieved an objective response then a total of 46 patients would be recruited.

Kaplan–Meier survival curves were compared using log-rank tests. Statistical analyses were performed using SPSS version 17.0 (IBM corporation, Armonk, NY, USA). The efficacy analysis was performed on the intention-to-treat population, which comprised all patients who received at least two cycles of study treatment.

### Ethics

The study was carried out fully in accordance with the requirements of Good Clinical Practice and the Declaration of Helsinki. The protocol was approved by the ethics committees of our institution. Patients were informed of the investigational nature of the study and provided written informed consent before registration. The trial was registered at clinicaltrials.gov (NCT01311050).

## Results

### Patients

Fifty-four patients were entered into the Phase I and Phase II studies between 24 January 2009 and 14 December 2011. One patient was found at a later stage to have a concurrent chaolangiocarcinoma, rather than metastatic colon cancer and was excluded from analyses other than the Phase I toxicity evaluation. All other analyses included the remaining 53 patients.

The study population was roughly equally divided between men and women (Table1). The majority of patients had ECOG PS 2, with tumors in the colon or rectosigmoid. About half had undergone surgery, a minority had previously received adjuvant chemotherapy, but none had received radiotherapy. Similar numbers of patients had single or multiple metastases, most commonly in the liver. Wild-type K-ras or K-ras mutations were also found in similar numbers of patients.

**Table 1:** Patients at baseline (*n* = 53)

Median age (range), years	52 (23–74)
Male/female, *n* (%)	29 (55)/24 (45)
ECOG performance status, *n* (%)	
0	7 (13)
1	35 (66)
2	11 (21)
Primary tumor site, *n* (%)	
Colon	23 (40)
Rectosigmoid	21 (36)
Rectum	9 (15)
Prior surgery for primary tumor, *n* (%)	29 (55)
Prior adjuvant chemotherapy, *n* (%)	6 (11)
Prior radiotherapy, *n* (%)	0
Number of metastasis sites, *n* (%)	
Single	22 (42)
Multiple sites	31 (58)
Metastasis sites, *n* (%)	
Liver	35 (66.0)
Lung	22 (41.5)
Lymph nodes	21 (39.6)
Peritoneum	14 (26.4)
K-ras, *n* (%)	
Wild-type	20 (37.0)
Mutated	20 (37.0)
Unknown	13 (26.0)

All data are given as *n* (%), except where indicated. ECOG, Eastern Cooperative Oncology Group.

### Maximum tolerated dose of irinotecan in the Phase I study

Three patients were received irinotecan at a dose of 150 mg/m^2^, of whom one developed Grade 4 diarrhea and fatigue in cycle 2. Three further patients received irinotecan 200 mg/m^2^, of whom one developed Grade 3 diarrhea and neutropenia and one developed Grade 3 vomiting. The maximum tolerated dose of irinotecan was therefore 150 mg/m^2^. Recruitment commenced for the Phase II trial using this dose level and an additional 47 patients were enrolled. However, a high incidence of Grade 3 and 4 toxicity (mainly diarrhea) led to a reduction in the capecitabine dose to 800 mg/m^2^ twice daily after 30 patients had been enrolled.

### Treatments

A total of 230 cycles of treatment were administered, with a median of five cycles per patient (range 1–8). Six patients received only one cycle of chemotherapy, either due to withdrawal of consent or Grade 4 toxicity. Thirty-four patients received the planned 5–8 cycles of induction triplet chemotherapy. Reasons for receipt of less than five cycles included toxicity in 11 patients, progression in four patients, withdrawn consent in three patients, and temporary loss of follow-up in a further patient. The relative dose intensity was 92% of that planned for irinotecan and oxaliplatin, and 79% of that planned for capecitabine. Maintenance treatment with capecitabine and bevacizumab was administered to 32 patients (60%).

Surgical resection of metastatic disease (Table2) was attempted with a curative intent in 13 patients (24.5%): four (7.5%) had surgical resection of the primary tumor, three (11.3%) had liver resection only, and six (11.3%) underwent cytoreductive surgery with hyperthermic intraperitoneal chemotherapy. Radical (R0) resection was achieved in 10 patients (18.9%) with a pathological complete response (pCR) in two of these patients (each had pCR of the primary tumor and of the metastasis in the liver or peritoneum).

**Table 2:** Surgery of metastases

	*N* (%)
Surgical resection	
Yes/no	13 (25)/40 (75)
Margin: R0/R1	10 (19)/3 (6)
Type of surgery	
Liver resection	3 (6)
CRS and HIPEC	5 (9)
Primary tumor resection and liver resection	3 (6)
Primary + CRS and HIPEC^1^	1 (28)
Lung resection	1 (2)

1 Primary: primary tumour resection, CRS and HIPEC: cytoreductive surgery and hyperthermic intraperitoneal chemotherapy.

### Efficacy

Of the 53 patients included in efficacy analyses, 45 were evaluable for response evaluation (four patients withdrew consent, two were discontinued for Grade 4 toxicity and two died). Two patients (4.4%) had a complete response and 27 patients (60%) had a partial response, for an overall response rate of 64.4%. Stable disease was observed in 31.1% and progressive disease in 4.4%. Fifteen patients (33.3%) achieved a response at the first evaluation (early treatment response) and 27 (60%) achieved early tumor shrinkage. The time to best response was 48 days (range 18–1041) and the median DpR was 33% (range −12 to 100).

**Figure 1:**
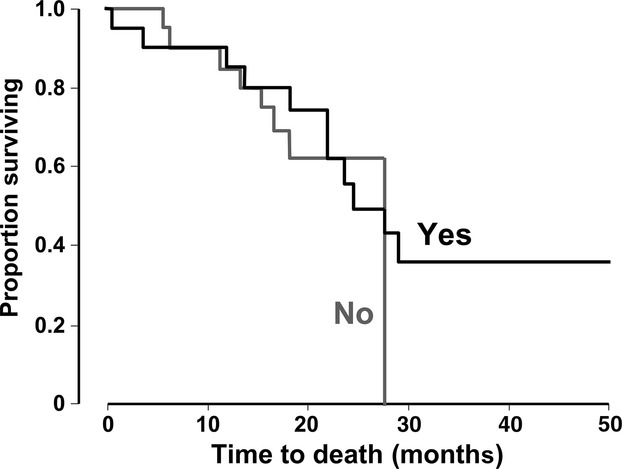
Kaplan–Meier estimates of survival in 53 patients.

**Figure 2:**
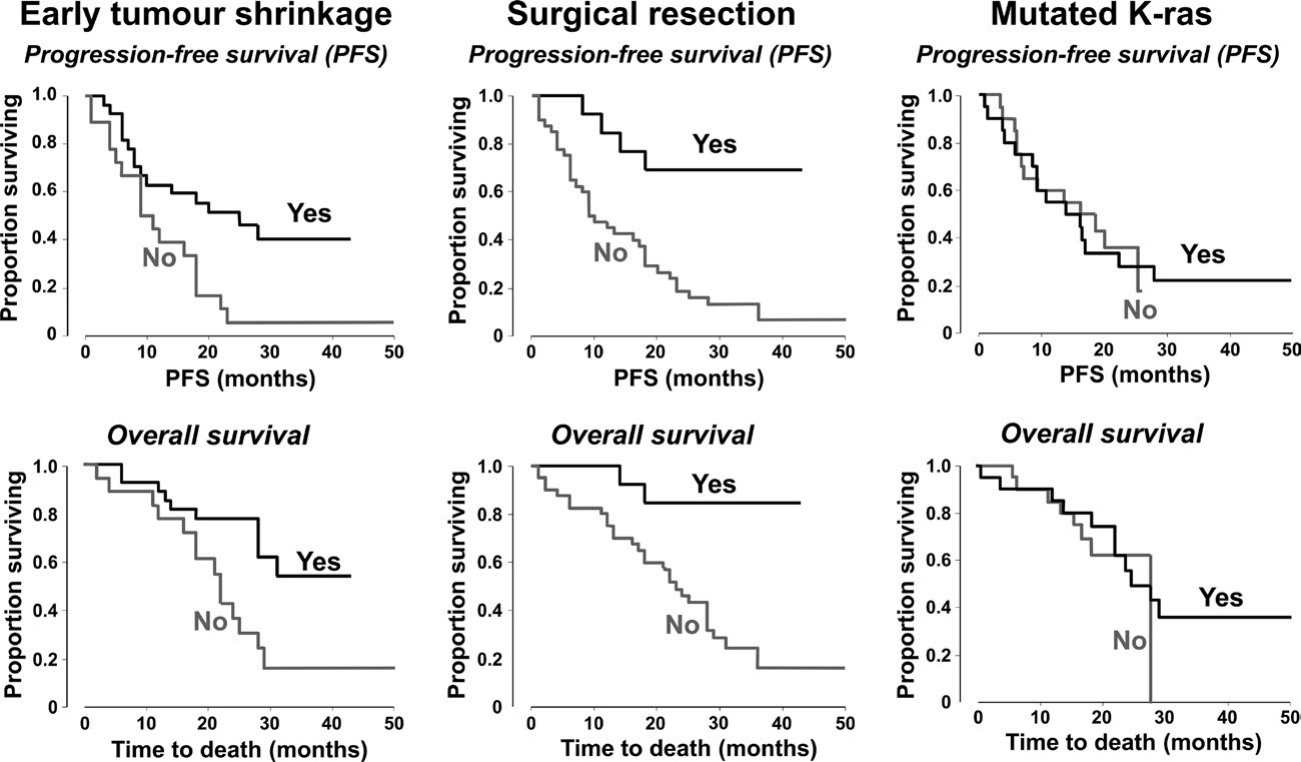
Kaplan–Meier estimates of progression-free survival and overall survival in subgroups of patients defined according to early tumor shrinkage (in 43 patients), surgical resection (in 53 patients), or K-ras mutation status (in 40 evaluable patients).

The median follow-up duration was 28 months (range 1–50; 95% CI 23–33), at which time 39 patients (74%) had progressed. Median PFS was 16 months and median OS was 28 months in the overall population (Table3, Fig.1). Median PFS was significantly longer in patients with early treatment response or early tumor shrinkage, or in subjects who underwent surgery with curative intent (Table3, Fig.2). OS was significantly prolonged in subjects with early tumor shrinkage or surgical resection. K-ras mutations did not influence PFS or OS (Table3, Fig.2).

**Table 3:** Median survival in selected subgroups

Patients	PFS (months)	P	OS (months)	P
All (*n* = 53)	16 (10.6–21.4)	–	28 (23.2–32.7)	–
Early response (*n* = 15)	28 (16–41)	0.024	28 (18.7–37.3)	0.55
No early response (*n* = 38)	9 (6–12)		28 (22.6–33.3)	
Early tumor shrinkage (*n* = 27)	25 (12–38)	0.006	Median not reached	0.006
No early tumor shrinkage (*n* = 26)	9 (4–14)		22 (20–24)	
Mutated K-ras (*n* = 20)	13.7 (2.6–24.8)	0.88	24.7 (17.0–32.4)	0.82
Wild-type K-ras (*n* = 20)	15.8 (6.8–24.8)		27.7 (23–32.6)	
Surgical resection (*n* = 13)	Median not reached	0.001	Median not reached	0.002
No surgical resection (*n* = 40)	9 (5–19)		23 (18–28)	

Figures in parentheses are 95% CI. PFS, progression-free survival; OS, overall survival.

### Toxicity

All 53 patients were evaluable for toxicity (Table4). Diarrhea was the most common Grade 3/4 toxicity was of diarrhea (36%), which was complicated by dehydration and renal impairment in one patient, and by neutropenia (32%), which was complicated by fever in nine patients. Other grade 3/4 adverse events were nausea/vomiting (21%), fatigue (17%), anemia (6%), thrombocytopenia (4%), and allergic reaction (4%). Grade 4 arterial thrombosis was reported in one patient who developed cerebrovascular thrombosis. Grade 3 peripheral neurotoxicity was reported in one patient.

**Table 4:** Toxicity (*n* = 53)

	Number with toxicity (%)			
	Grade 1	Grade 2	Grade 3	Grade 4
Anemia	8 (15)	9 (17)	3 (6)	0
Neutropenia	0	7 (13)	16 (30)	1 (2)
Thrombocytopenia	2 (4)	0	0	2 (4)
Febrile neutropenia	0	0	9 (17)	0
Hand and foot syndrome	17 (32)	8 (15)	0	0
Nausea/vomiting	11 (21)	24 (45)	10 (19)	1 (2)
Anorexia	0	7 (13)	3 (6)	0
Fatigue	4 (8)	12 (23)	7 (13)	2 (4)
Mucositis	21 (40)	3 (6)	0	0
Diarrhea	2 (4)	23 (43)	18 (34)	1 (2)
Peripheral neuropathy	20 (38)	24 (45)	1 (2)	0
Allergic reaction	1 (2)	1 (2)	2 (4)	0
Skin reaction	1 (2)	0	0	0
Hypertension	0	4 (8)	0	0
Arterial thrombosis	0	0	0	1 (2)
Venous thrombosis	0	0	0	0
Bowel perforation	0	0	0	0
Others	1 (2)	9 (17)	8 (15)	2 (4)

Toxic death occurred in three patients, two after the first cycle and one after fifth the cycle of chemotherapy. All deaths occurred outside our institution; one developed fever at home and refused to go to a hospital, one death was secondary to cerebrovascular accident and one died from non-neutropenic septic shock. There was no significant difference in toxicity between patients who received capecitabine at a dose of 1000 or 800 mg/m^2^.

## Discussion

The result of this trial are consistent with the results of previous randomized trials that demonstrated enhanced efficacy of the ripple-therapy regimens in mCRC 7,8. The response rate in our trial of 60% is similar to the roughly 60–80% response rates observed with other triple-therapy regimens 7,8,20,21,27,28. The PFS obtained in our regimen (16 months) represents one of the best reported results so far in mCRC, although this did not translate to a similarly high OS (28 months). Receipt of no more than five cycles by half of the patients and the high toxicity of the regimen may account for this finding.

We observed marked toxicity, with Grade 3 and 4 diarrhea (35%) requiring frequent dose reduction, especially of capecitabine, resulting in a low relative dose intensity for capecitabine of 79%, although the dose reduction did not translate into a reduced frequency of Grade 3/4 diarrhea. Febrile neutropenia was also common (17%), compared with other evaluations of triple regimens in mCRC 7,8,20,21,27,28. It is apparent that the planned dose intensity of both bi-weekly triplet regimens reported for capecitabine (5000 mg/m^2^ per week 20 and 7000 mg/m^2^ per week 21) was lower than in our trial, even after the capecitabine dose was reduced to 800 mg/m^2^ twice-daily (7466 mg/m^2^ per week). This probably explains the difference in toxicity between our study and these earlier trials. In addition, clinical experience in the Middle East suggests that the tolerance of Saudi patients to standard doses of capecitabine may be lower than that reported for Western populations. The toxicity encountered with our regimen might also be explained in part by 87% of our patients having PS 1–2. Nevertheless, other regimens combining capecitabine and irinotecan have yielded similar high rates of toxicity, with incidence rates for diarrhea approaching 40% 18,19. These regimens maintained activity with decreased toxicity by lowering the doses of both irinotecan and capecitabine 29,30.

We also demonstrated that the concept of early tumor shrinkage, reported previously, is not limited to anti-epidermal growth factor receptors (EGFR) regimens 26,31,32. Patients who achieved ≥20% tumor shrinkage at the first evaluation (after two cycles of induction therapy) had improved PFS and OS compared with patients who did not. Our study also confirmed the lack of influence of K-ras mutation status on prognosis in patients treated with a bevacizumab-containing triple-chemotherapy regimen, as reported elsewhere 27,33,34.

It has been reported that surgery for resectable metastasis improves survival in patients with mCRC 35–37, and our data provide further confirmation of this benefit. In addition, our study confirms the high rate of R0 resection (approaching 19%) in patients treated with triple-chemotherapy regimens. The high percentage of surgical resection confirms the feasibility of such procedures in patients receiving bevacizumab containing regimens.

The main limitations of our study were that it was a Phase I/II noncomparative trial, conducted at a single institution, and in a relatively small patient population. On the other hand, our trial enrolled an unselected patient population who were similar to the patients we see in daily practice, as shown by the relatively high percentage of patients with PS 2 (20%) and multiple organ involvement (58%).

In conclusion, the three weekly triple-chemotherapy regimen of capecitabine, oxaliplatin, and irinotecan, combined with bevacizumab, was active in the first line treatment of mCRC, although at the expense of a high level of toxicity. We do not recommend further application of this regimen at the doses described above. Further evaluation of this regimen in a more selected group of patients with mCRC with better PS and an adjusted dose of capecitabine and irinotecan may yield lower toxicity while maintaining therapeutic activity.
